# A Rare Osteoid Forming Carcinosarcoma Ex-Pleomorphic Adenoma of the Parotid Gland

**DOI:** 10.1155/crip/7588391

**Published:** 2025-08-26

**Authors:** Nyein Nyein Htun, Daniel Nguyen, Beverly Y. Wang, Anoosh Montaser, Behdokht Nowroozizadeh

**Affiliations:** ^1^Department of Pathology and Laboratory Medicine, University of California, Irvine, California, USA; ^2^Department of Radiology, University of California, Irvine, California, USA

**Keywords:** carcinosarcoma, osteoid formation with osteoclasts giant cells, parotid gland, preexistent pleomorphic adenoma, radiation exposure

## Abstract

Salivary gland carcinosarcoma is a combination of malignant epithelial and sarcomatous tumors and can develop from a preexisting pleomorphic adenoma or de novo. These tumors are rapidly growing infiltrative tumors and have an extremely poor prognosis, with a high frequency of lymphatic and hematogenous spread at the time of diagnosis. Approximately half of the cases of carcinosarcoma arise from preexisting pleomorphic adenoma with a long-standing clinical history of parotid mass. The carcinomatous component is most commonly squamous cell carcinoma or adenocarcinoma, while chondrosarcoma is the most frequent sarcomatous component. Our case is particularly unusual due to the presence of osteosarcomatous differentiation as the sarcomatous component. In addition to its histological rarity, our patient has never been reported a prior parotid mass or history of pleomorphic adenoma in this location. However, thorough examination of the radical parotidectomy specimen revealed sclerosed foci of pleomorphic adenoma in addition to carcinosarcoma with osteoid formation. In conclusion, we report an unusual case of carcinosarcoma ex-pleomorphic adenoma with osteoid formation and osteoclast giant cells in a patient without a history of pleomorphic adenoma or parotid mass.

## 1. Introduction

Salivary gland carcinosarcoma is a combination of malignant epithelial and sarcomatous tumors and can develop from preexistent pleomorphic adenoma (PA) or de novo. PA is a benign mixed tumor of the salivary gland and commonly occurs in the parotid gland (~70%–80%), followed by the oral cavity and submandibular gland [1]. PA usually presents as a slow-growing, painless mass, but on rare occasions can manifest in rapid growth of the mass and facial nerve symptoms (in cases of parotid gland origin), suggestive of a malignant transformation. The rate of malignant transformation in recurrent PA was 0%–23% [2]. The histology type of the carcinoma component can be of any type: most commonly it is salivary duct carcinoma, followed by myoepithelial carcinoma and adenocarcinoma not otherwise specified (NOS) [3]. The combined salivary gland tumor (carcinosarcoma) is a very rare tumor that accounts for < 0.5% of all malignant salivary gland tumors. Carcinosarcoma from preexistent PA is an aggressive tumor, and approximately 11 cases have been reported in the literature. The carcinomatous component of carcinosarcoma is most commonly squamous cell carcinoma or adenocarcinoma, whereas the most common sarcomatous component is chondrosarcoma [4]. Carcinosarcoma ex-PA with osteoid formation and osteoclastic giant cell cases were rarely reported. We herein present an unusual case of salivary gland carcinosarcoma ex-PA which was composed predominantly of osteosarcoma and osteoclast giant cells, along with fine needle aspiration (FNA) cytology and immunohistochemical findings.

## 2. Case Presentation

We report a case of a mid-80s female patient who presented to our hospital with a chief complaint of pain localized to the left jaw and temple, with a history of a growing and painful left parotid mass. The pain progressed rapidly, starting 2 months ago from the left jaw and radiating to the left orbit and forehead. Her past medical history did not include any long-standing mass or prior tumor in the parotid area.

Her past medical history was significant for a resected squamous cell carcinoma of the vocal cord more than 20 years ago, a resected low-grade neuroendocrine tumor of the gastric antrum 12 years ago, a radiated inflammatory myofibroblastic tumor of the bladder, and multiple skin cancers including squamous cell carcinoma and basal cell carcinoma of the bilateral upper extremities.

**Figure 1:**
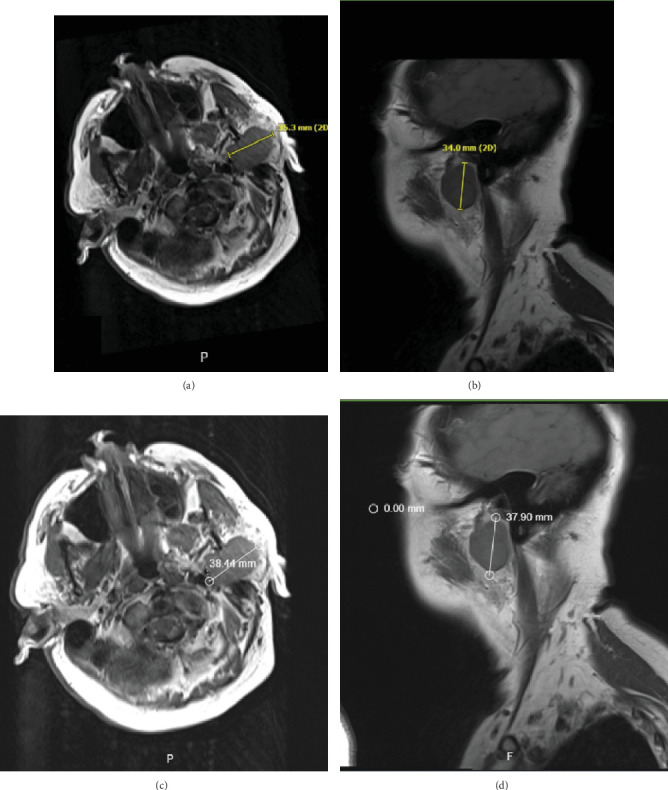
MRI soft tissue neck axial and sagittal T1 weighted images. (a, b) Left parotid mass at the time of initial workup of left jaw pain. (c, d) Rapid growth of parotid mass 10 days after initial diagnosis.

**Figure 2:**
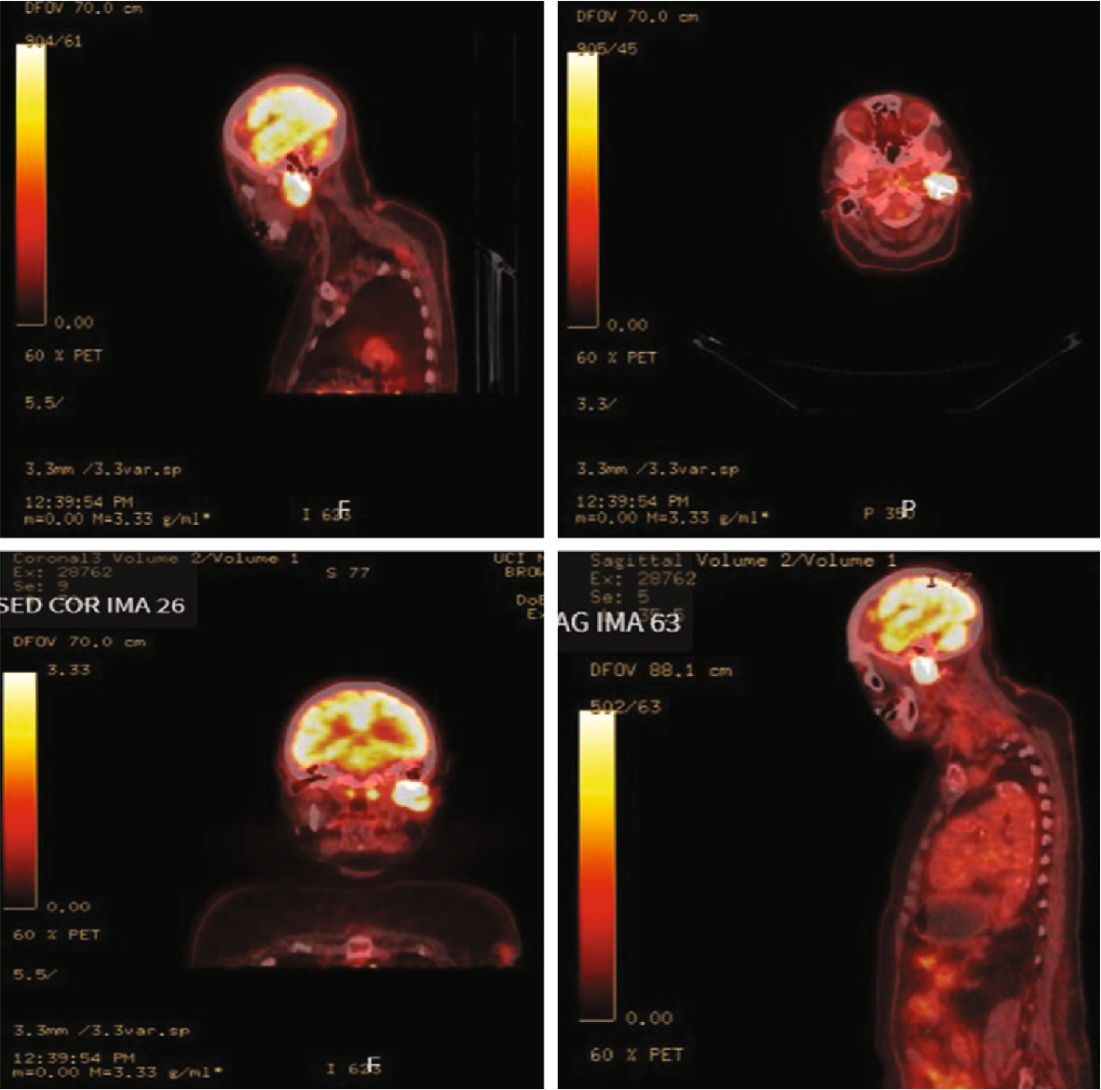
PET/CT image showed a hypermetabolic left parotid mass compatible with a known neoplasm involving both the superficial and deep parotid lobes, with activity measuring up to 12.0 SUV. No significant FDG-avid lymphadenopathy.

Physical examination revealed a palpable parotid mass with numbness and decreased sensation on the left side of the face. The face was symmetric, with CN VII grossly intact. Initial magnetic resonance imaging (MRI) investigation revealed a 3.5 cm left parotid mass extending to the stylomastoid foramen, suggestive of a malignant lesion (Figure 1). Positron emission tomography/computed tomography (PET/CT) revealed a left parotid mass compatible with a primary neoplasm without definite evidence of FDG-avid nodal or distant metastatic lesions (Figure 2).

The second MRI performed 10 days after the initial MRI again showed a left parotid mass measuring up to 3.8 cm, involving both the superficial and deep parotid lobes, the mandibular fossa, and retrograde perineural involvement of the mastoid facial nerve segment, consistent with the result of first MRI (Figure 1c,d).

**Figure 3:**
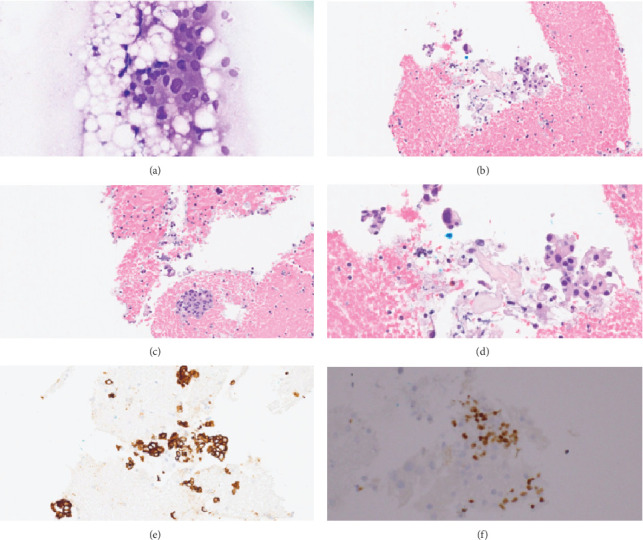
FNA of the parotid mass showed carcinoma with glandular differentiation, Milan Category VI. (a) Smear preparation of FNA 400X. (b, c) Cell block preparation 200X. (d) Cell block preparation 400X. (e) CK7 immunopositivity 200X. (f) P63 immunopositivity 200X.

The FNA investigation revealed pleomorphic epithelial cells with expressions of CK7 and P63. A diagnosis of carcinoma with glandular differentiation, Milan Category VI, was rendered based on FNA findings (Figure 3).

The patient then underwent radical parotidectomy and neck dissection.

**Figure 4:**
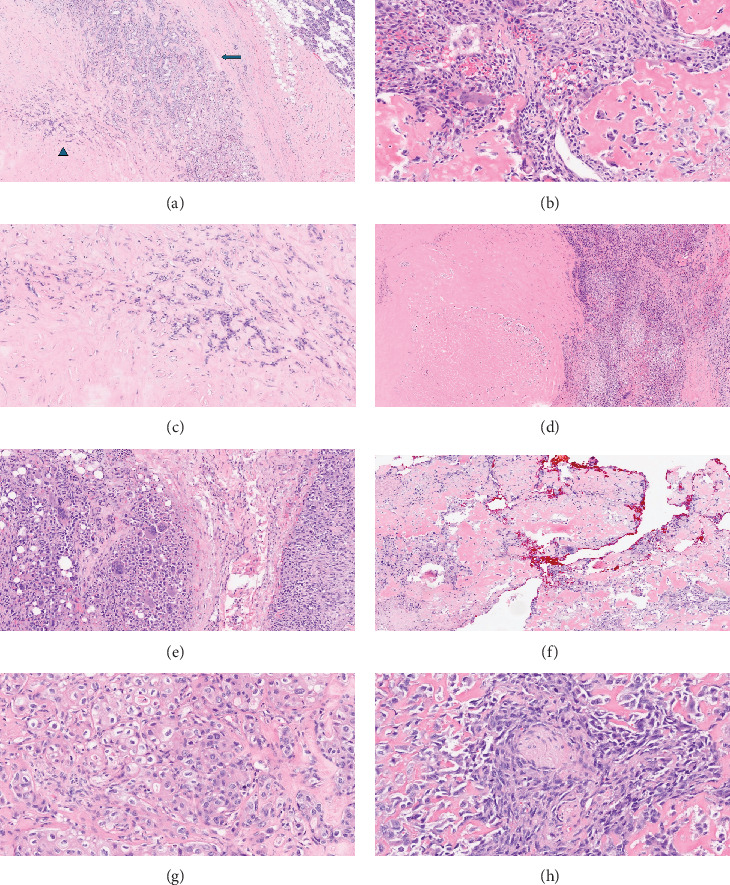
Carcinosarcoma ex-pleomorphic adenoma. (a) Carcinoma component (arrow) with pleomorphic adenoma (arrowhead) and surrounding normal parotid gland 40X. (b) Osteosarcoma with tumor osteoid formation 200X. (c) Preexistent sclerosed pleomorphic adenoma 100X. (d) Tumor necrosis in the sarcomatous area 40X. (e) Osteosarcoma with osteoclast-like giant cell formation 100X. (f) Resection margin (inked with red color) involvement by sarcomatous component 40X. (g) Adenocarcinoma NOS 200X. (h) Carcinosarcoma with perineural invasion 200X.

The pathology report from the radical parotidectomy and neck dissection revealed a 4 cm carcinosarcoma of the parotid, with mixed adenocarcinoma and osteosarcoma, along with tumor necrosis (about 10% of tumor area) and atypical mitoses. Numerous osteoclast giant cells were identified in the sarcomatous areas, the osteosarcomatous area being the predominant component of the tumor, approximating 60% of the entire tumor. There were sclerotic foci of preexisting PA, which were abruptly transitioned to adenocarcinoma. Perineural invasion and lymphovascular invasion were identified, with negative nodal disease (Figure 4).

**Figure 5:**
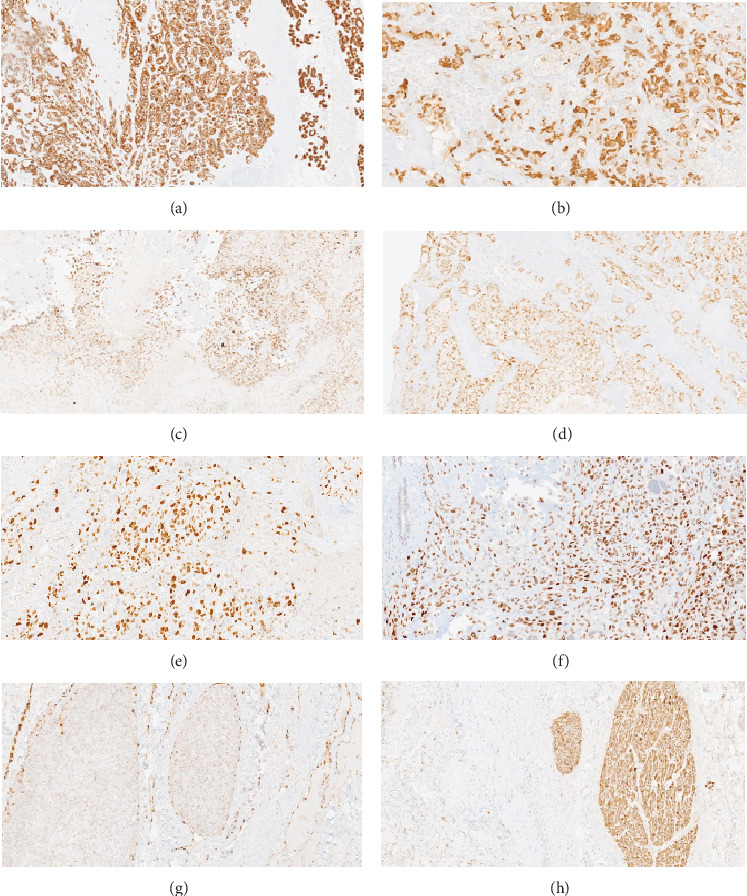
(a) CK7 membranous stain positive in adenocarcinoma areas 100X. (b) Mammaglobin cytoplasmic and membranous stain (positive) 100X. (c) SATB2 (nuclear stain) positive osteosarcoma 100X. (d) GATA3 nuclear stain positive 100X. (e) Ki-67 proliferation index 100X. (f) P53 nuclear staining aberrant type 100X. (g) Lymphovascular invasion identified by ERG 100X. (h) Tumor cells are negative for S100, but nerves are highlighted by S100 40X.

Immunohistochemically, the carcinoma components were positive for AE1/AE3, CK7, GATA3, and mammaglobin, while the osteosarcoma areas were immunoreactive for SATB2 with tumor osteoid formation. The tumor cells aberrantly expressed P53, and the Ki-67 proliferation index was more than 30% (Figure 5).

The surgical resection margin (inked margin) was positive for sarcomatous components, and the tumor extended to the periparotid adipose tissue. The diagnosis of carcinosarcoma ex-PA with mixed adenocarcinoma and osteosarcoma was rendered.

## 3. Discussion

Carcinoma ex-pleomorphic adenoma (CXPA) is an epithelial and/or myoepithelial malignancy that presents in association with a primary or recurrent PA [4]. Most CXPAs arise in the parotid gland, but they may also originate from the submandibular gland or minor salivary glands. CXPA accounts for 3.6% of all salivary gland tumors and 12% of all salivary gland malignancies [5]. People with CXPAs are, on average, at least one decade older than patients with PA [6]. The tumor is slightly more common in women.

The main presentation is a long-standing painless mass with recent rapid progression or a previous diagnosis of PA. Occasionally, the tumor may be asymptomatic [3]. The majority of CXPAs present as irregular, lobular, and heterogeneously enhancing masses with uneven or partially uneven margins on CT and MRI, often with internal calcifications [7]. Multiple cervical lymph node and distant metastases are observed at the time of diagnosis of invasive CXPA [6]. On rare occasions, malignant transformation can be both carcinomatous and sarcomatous in nature.

The immunohistochemical profile of CXPA included positive staining reactions in the malignant component for AE1/AE3 in 97% of cases, CK7 in 94%, epithelial membrane antigen in 86%, carcinoembryonic antigen in 75%, vimentin in 52%, and S100 protein in 29%. Expression of p53 and c-erbB-2 oncoproteins was detected in 41% and 30% of the carcinomas, respectively [8]. FNA smears can show carcinoma components or may show PA, depending on the sampling. It is uncommon for both components to be identified on the cytology specimen, and necrosis in an otherwise PA may be the only clue, apart from a previous history of a long-standing mass or previous biopsy [9].

Most CXPAs are aggressive, with local and distant recurrences, and the 5-year survival rate ranges from 25% to 75%. Disease-specific mortality predictors include a tumor size of 4 cm or greater, multiple positive lymph nodes, and distant metastatic disease [10].

True malignant mixed tumors (carcinosarcomas) of salivary glands are rare, and one study hypothesized that carcinosarcoma almost always develops from PA, with a complex multistep adenoma–carcinoma–sarcoma sequence, based on two alternative histogenesis pathways [11, 12]. Most cases of carcinosarcoma manifest in major salivary glands (parotid, 70%; submandibular, 19%); minor gland occurrences are rare. The mean presentation age is 58 years, with no sex preponderance [4].

Half of the cases of carcinosarcoma arise from preexisting PA, but a few cases arise de novo. Extensive sampling combined with immunohistochemistry enabled the morphogenetic identification of PA in 93.8% of cases [12]. Irradiation may have been responsible for inducing a true malignant mixed tumor, distinct from the more common malignancy that may arise in PA, which is a simple carcinoma [13]. Irradiation to prior PA might have been involved in the pathogenesis of carcinosarcoma ex-PA [13].

Malignant mixed tumors are large, infiltrating tumors with frequent necrosis and hemorrhage. A small sclerotic nodule may represent a preexisting PA. The carcinomatous component is most commonly a squamous cell carcinoma or adenocarcinoma, whereas the most common sarcomatous component is a chondrosarcoma [4]. Immunohistochemical investigation is essential for distinguishing carcinosarcoma from other sarcomatous salivary gland tumors.

The cytological findings of carcinosarcoma ex-PA have been reported in very few cases. In one case study, various components, such as the presence of atypical epithelial cell clusters and singly scattered atypical cells with stromal components on cytological specimens, warranted consideration of the diagnosis of carcinosarcoma ex-PA [14]. However, carcinosarcomas can show clusters of large epithelial cells with moderate to abundant cytoplasm, large pleomorphic hyperchromatic nuclei, and prominent nucleoli, along with occasional giant cells and spindle cells showing atypical nuclei in a background of necrosis [15]. The FNA investigation of our patient's parotid mass before radical resection revealed carcinoma with glandular differentiation only, Milan Classification VI, without any evidence of stromal or sarcomatous components. The radical parotidectomy specimen revealed carcinosarcoma of the parotid gland, with the carcinoma components being adenocarcinoma, NOS, and sarcoma components consisting of osteosarcoma with osteoclast giant cells. The osteosarcomatous differentiation was confirmed by SATB2 immunoexpression. Cytologic examination of carcinosarcoma is challenging due to its overlapping cytomorphologic characteristics with other high-grade malignant salivary gland tumors [16, 17].

Our reported case was very peculiar for its unusual osteosarcoma with tumor osteoid formation. The prognosis of carcinosarcoma is extremely poor, with a high frequency of local recurrence and lymphatic and hematogenous spread. Mortality is 50%, and limited data are present in the literature for follow-up studies [4]. Our case had positive surgical margins with identifiable lymphovascular invasion and perineural invasion at the time of diagnosis. All the lymph nodes retrieved from the neck dissection were negative for nodal disease.

Three weeks after the initial diagnosis, the patient received radiation treatment and continued with chemotherapy another 3 weeks later. In subsequent CT imaging, there were 2.6 cm pulmonary masses with bilateral pleural effusion. Unfortunately, the patient did not tolerate the treatment, and the condition progressed despite intensive radical surgery, chemotherapy, and radiotherapy. Six months following the date of the FNA diagnosis, the patient's condition had significantly declined, and she eventually passed away.

## 4. Conclusion

This is a unique case report of carcinosarcoma ex-PA of the parotid gland with osteosarcoma as the sarcomatous component and extensive tumor osteoid formation in a patient with no known prior history of a parotid mass. Additionally, the tumor was aggressive with p53 overexpression. Very few cases of osteosarcoma from carcinosarcoma ex-PA have been reported, with osteoid formation. From a cytopathology standpoint, it is especially important to note the pitfalls of cytologic examination, as sarcomatous components and remaining PA components can easily be missed on FNA specimens due to sampling artifacts.

## Data Availability

Research data are not shared.
